# Supervised model based polycystic ovarian syndrome detection in relation to vitamin d deficiency by exploring different feature selection techniques

**DOI:** 10.1038/s41598-025-14728-z

**Published:** 2025-08-26

**Authors:** A. Archana, V. Sumathi

**Affiliations:** 1 School of Electrical Engineering, Vellore Institute of Technology, Chennai, Tamil Nadu India; 2 Centre for E-Automation Technologies, School of Electrical Engineering, Vellore Institute of Technology, Chennai, Tamil Nadu India

**Keywords:** PCOS, Vitamin D3, Feature selection techniques, ML models, Health care, Signs and symptoms

## Abstract

Due to urbanization and modern lifestyle, most of women in today’s world are prone to Polycystic Ovarian Syndrome (PCOS), which is a hormonal disorder. Though the symptoms shown by this disease are often uncared, it seriously affects the reproductive health of women. Early detection of PCOS helps in managing several other attributes that are closely related to it. This article aims to study the impact of Vitamin D3 in PCOS and non-PCOS individuals. The goal is attained by building a tailored dataset with 1368 records and 43 attributes. Initially, the acquired dataset is pre-processed by handling missed values, outlier detection and data balancing by employing Probabilistic Principal Component Analysis (PPCA), Interquartile Range (IQR), Z-score standardization and SMOTE respectively. The significant features are selected by exploring different approaches such as filter based (Chi-Square, ANOVA), wrapper based (Electric Eel Foraging Optimization Algorithm) and embedded methods (LASSO, XGBoost). The selected features are utilized to train classifiers such as Random Forest (RF), k-Nearest Neighbour (k-NN), Decision Tree (DT) and Support Vector Machine (SVM). The experimental results show that the performance of EEFOA with RF prove the best accuracy rates of 98.8% with a F-measure of 98.19%. Explainable Artificial Intelligence (XAI) techniques such as SHAP and LIME are then employed to showcase the feature importance. It is observed that over 40% of PCOS patients are affected by deficiency and insufficiency of vitamin D3.

## Introduction

Polycystic Ovary Syndrome (PCOS) is a hormonal disorder being confronted by 5% to 10% of women population of reproductive age is linked to reproductive complications and metabolic disorders. This disorder is recognized as a significant contributor to chronic anovulation and anovulatory infertility^1,2^. Usually, this syndrome is diagnosed by fundamental biochemical and clinical markers, in addition to ultrasonography. The clinical manifestations of PCOS differ by age, with patients displaying a range of symptoms including menstrual irregularities, obesity, dysfunction of ovaries, hyperandrogenism with hirsutism in certain cases^3^. The etiology of this syndrome remains ambiguous, though the diverse susceptibilities among patients are certainly influenced by various environmental and genetic risk factors^4^.

The heterogeneity appears to be affected by multiple factors, such as Insulin Resistance (IR), uterine nutrition, premature adrenal gland hyperactivity, genetics, prenatal androgen sensitivity and body weight fluctuations^5^. The management of PCOS is crucial for addressing and mitigating enduring conditions linked to the disorder, including type 2 diabetes, obesity and other metabolic dysfunctions, as well as psychological issues^6^. This is necessary to alleviate both hyperandrogenism and hyperinsulinemia, as PCOS significantly increases the risk of these factors by at least 2 to 3 times in women^7^.

The most intriguing comorbidities examined in women with PCOS are vitamin and mineral deficiencies. Consequently, supplementation with vitamins or minerals exerts advantageous effects in the management of comorbidities associated with PCOS, such as anovulation, obesity or elevated BMI, hyperinsulinemia, cardiovascular disorders, heightened androgen levels, as well as mental and psychological issues^8,9^.

Vitamin D is a quintessential fat-soluble vitamin that plays the role of antecedent for vital hormones to regulate parathyroid hormone, phosphate and calcium metabolism, which are necessary for growth and development of bones, modulation of immune response and protein synthesis^10^. Vitamin D encompasses both vitamin D2 (ergocalciferol) and vitamin D3 (cholecalciferol), which can be sourced from luminous plant materials and sunshine, respectively. Ergocalciferol (vitamin D2) is metabolized into the biologically active form 25(OH)D (calcidiol), which indicates the blood level of the antecedent to the active steroid hormone^11^.

Research indicates that blood concentrations of 25(OH)D below 20 ng/mL are correlated with metabolic syndrome, highlighting the significant relationship between Vitamin D Deficiency (VDD) and PCOS, as well as its detrimental effects on health^12^. Reduced levels of 25(OH) D elevate the risk of cardiovascular diseases and intensify symptoms of PCOS, such as IR, hyperandrogenism, ovulatory irregularities, infertility and obesity^13^.

In PCOS affected women, vitamin D influences metabolism and individuals with PCOS exhibit a higher prevalence of vitamin D insufficiency^14^. Recent studies indicate that VDD is prevalent among population, potentially correlating with metabolic and endocrine disorders associated with PCOS ^15^. The importance of Vitamin D as nutritional supplement for the management of PCOS in afflicted women is an emerging notion.

Recognizing the reputation of vitamin D in reproductive health, this article tends to analyse the impact of vitamin D over PCOS patients. Modern urbanized lifestyle seriously influences the reproductive health of Indian women and this work tends to emphasize the importance of vitamin D to manage PCOS. The major highlights of this work are listed below.

- A multitude of studies have examined the associations between vitamin D level and PCOS, as well as metabolic and hormonal dysfunctions specifically related to PCOS; nonetheless, the relationship remains ambiguous.
- Despite an increasing number of intercession studies evaluating the benefits of vitamin D supplementation on PCOS, there is a deficiency of compelling indication establishing a causal relationship between vitamin D levels and PCOS, primarily attributable to small sample sizes.
- This work is carried out on 1368 records of PCOS, whereas most of the existing works deal with only 541 records.
- This work makes effort to pre-process the dataset by dealing with missing data, outlier detection and data balancing by employing PPCA, IQR, Z-score normalization and SMOTE.
- Various feature selection methods such as filter based (Chi-square, ANOVA), wrapper based (Electric Eel Foraging Optimization Algorithm) and embedded based (LASSO, XGBoost) techniques are explored and experimented by this work.
- Multiple classifiers such as Decision Trees (DT), k-Nearest Neighbour (k-NN), Support Vector Machine (SVM) and Random Forest (RF) are trained with the selected features.
- RF with EEFOA is proven to showcase the best result with accuracy and F-measure rates of 98.8% and 98.19% respectively.
- XAI techniques such as SHAP and LIME are incorporated to showcase the importance of features for better interpretation of ML model.

The aim of this work is to quantitatively synthesize existing evidence to ascertain whether vitamin D levels are diminished in women with PCOS relative to those without PCOS. The subsequent sections of this article are structured as follows. Section “Review of literature” examines the literature about the PCOS classification, correlation between vitamin D and PCOS. The proposed work is detailed in Sect. “Proposed analytical model to study the association of vitamin D and pcos**”** and its performance is validated in Sect. “Results and discussion”, while the article concludes at Sect. “Conclusions”.

## Review of literature

This section intends to study the existing literature that analyses the relation between vitamin D and PCOS.In recent years, Machine Learning (ML) models have become increasingly significant in the field of medicine for the purpose of disease prediction.

In the study referred to as^16^,the authors assess the effectiveness of multiple ML models in predicting VDD in a group of thirty-four pupils. Independent factors such as age, weight, and sun exposure were taken into consideration by the researchers looking at the data. One of the classifiers that the authors used was RF, which stood out because to its high accuracy of 96% on test sets. The authors used numerous other classifiers. The k-NN and the RF classifiers were also applied in this study. According to the technique that was applied in this work, ML models have the potential to be a useful instrument for the prediction of VDD.

In^17^, the researchers looked at whether there was a correlation between the levels of vitamin D in individuals who had suffered an acute ischemic stroke and the clinical histories of those patients during their study. Logistic Regression (LR) and Extreme Gradient Boost (XGBoost) were two of the ML approaches that the researchers applied to conduct an analysis of the data that was acquired from 328 patients. The data was collected from medical professionals. An insufficient amount of vitamin D was discovered to be associated with an increased likelihood of undesirable outcomes, as indicated by the data. The application of LR produced an Area Under the Curve (AUC) value of 0.746, which is more specific than the previous outcome.

Participants in southern Bangladesh who had been diagnosed with systemic lupus erythematosus were the subjects of the research that was reported in^18^, which investigated the impact of VDD on these individuals. The goal of the study was to examine the risk factors that are associated with low vitamin D levels in that group of patients. This was accomplished by way of the gathering of clinical and demographic data from fifty patients, as well as the application of RF and Linear Regression analysis. With the use of these models, it was feasible to determine the significant variables that were responsible for predicting vitamin D levels. Haemoglobin and age are two examples of the factors that fall within this category. Because of its ability to deal with collinearity and interactions between variables, the RF model, which was developed through cross-validation, stood out as a particularly noteworthy model. Root Mean Square Error (RMSE) was 2.98, while Mean Absolute Error (MAE) was 2.68. Both values were obtained by the system.

In the study that was published in^19^, the scientists investigated the efficacy of various regression models for VDD prediction in a sample of individuals who were both obese and diagnosed with hypertension. It was necessary for them to use data from 221 patients to construct LR as well as two ML algorithms (LASSO and ElasticNet. Additionally, the significance of parameters such as blood pressure and levels of LDL cholesterol was brought to light in the process of predicting VDD. The ML models had improved performance in comparison to the normal LR, as demonstrated by a lower classification error rate and a higher AUC. This is in contrast to the LR, which had a value of 0.64.

A study was conducted by the authors of^20^ to evaluate the potential strategies that may be utilized to forecast VDD in persons who have hypertension. All the information that was used in the study was gathered from a total of one hundred and two patients who were treated at a university hospital in Spain. Different algorithms, such as LR and SVM, were utilized for data analysis. In order to effectively identify those individuals who were at a greater risk of VDD, the models were able to make use of a combination of feature selection strategies and classification algorithms. The SVM technique was found to be superior in terms of sensitivity, achieving a score of 98% throughout the evaluation process.

An investigation into the application of ML algorithms for the aim of assessing the levels of vitamin D in individuals was carried out by the authors of^21^. Ordinal LR (Elastic-net), SVM, and Regression Functions were applied in this work to do an analysis on the data that was gathered from 481 patients who were treated at a private hospital. As a result of the findings, it was determined that RF performed significantly better by achieving an accuracy of 94% and a F1 score of 0.95. The fact that RF was able to successfully analyse insignificant data samples was proved by this.

In^22^, various ML methods were applied to a total of 350 instances to evaluate VDD. During this research, a number of algorithms, including LR, SVM, RF, and OptRF, were among those that were examined and contrasted together. When the accuracy of OptRF was compared to that of SVM (64%) and LR (73%), it was discovered that OptRF showed a higher level of accuracy (91.42%). With the purpose of determining whether there was a correlation between the levels of vitamin D and neurological problems in patients who had experienced a cerebral infarction, the researchers who worked on the study ^23^investigated the matter. During the research, a total of two hundred patients were utilized for the purpose of training LR algorithms and XGBoost as predictive models. The scientists decided to look into the levels of VDD, cholesterol levels, and blood pressure as the variables that they wanted to study. The XGBoost algorithm displayed superior performance in comparison to the LR algorithm after being adjusted by cross-validation (70–30% training and testing, respectively). The AUC that the XGBoost method attained in the test set was 0.786, which was much greater than the AUC that the LR algorithm achieved, which was 0.761.

The authors of^24^ investigated the prediction of VDD in elderly individuals by making use of ML models of their own. In the course of the research, information was gathered from a total of 5106 persons, with 65 percent of them being picked at random for the purpose of training, and the remaining 35 percent being selected for testing. In contrast to a reference model that was constructed on the basis of LR, the models that were constructed included Lasso Regression, Elastic Net, RF, and DT. The ML models delivered results that were equivalent in terms of accuracy when it came to anticipating 25(OH)D levels that were lower than 50 nmol/L. On the other hand, these models performed much better than the reference model when it came to predicting levels that were lower than 25 nmol/L. It was determined that elastic regression achieved the highest AUC of 0.93.

The authors of^25^suggested a model for the prediction of PCOS in fertile patients. They employed an open-source dataset consisting of 541 patients from Kerala, India. The authors utilized ML models, such as AdaBoost, KNN, DT, NB, LR, RF, Extra Trees, SVM, and Gradient Boost for PCOS prediction. Additionally, they recommended the implementation of multi-stacking ML. In order to render model predictions comprehensible, interpretable, and dependable, they implemented Explainable AI methodologies. The results suggested that multi-stacking ML obtained the highest accuracy of 98%.

In^26^, a predictive model for the self-diagnosis of PCOS is developed using ML techniques. They utilized a publicly accessible Kaggle PCOS dataset that encompassed 44 features and contained data from 541 patients from 10 different hospitals in Kerala. The CatBoost classifier was implemented by the authors, who assessed its efficacy through K-fold validation. The classifier achieved an 82.5% accuracy rate for invasive procedures and a 90.1% accuracy rate for non-invasive clinical indicators. In^27^, a diverse array of ML techniques, such as LR, RF, SVM, CART, and naïve bayes classification, are employed to identify PCOS patients. The RF algorithm demonstrated exceptional performance in the diagnosis of PCOS on the tested dataset, obtaining a 96 percent accuracy rate.

In their investigation of the Kaggle PCOS dataset, the authors of^28^ implemented classical and ensemble classifiers to diagnose PCOS. They evaluated the advantages of feature selection techniques and ensemble classifiers. Furthermore, the efficacy of numerous classifiers, such as AdaBoost, RF, Extra Tree and Multi-Layer Perception was evaluated using the dataset with all features and reduced feature sets generated by different feature selection techniques. The RF classifier surpassed other classifiers by employing a restricted data subset derived from the integrated feature selection method, with an accuracy of 98.89%.

Inspired by these existing works, this article tends to present an analytical model for the sake of studying the association of vitamin D and PCOS, which is elaborated in the following section.

## Proposed analytical model to study the association of vitamin D and PCOS

The major goal of this work is to highlight the correlation of vitamin D and PCOS, which is accomplished by the key phases such as data acquisition and pre-processing, feature selection and PCOS predictive model. The data collection is challenging, as the public datasets are limited. The pre-processing step intends to handle missing values, while removing noisy and duplicate data. The dataset contains several features of the patient and hence, the appropriate features are selected by feature selection techniques. Finally, the ML models are trained by the selected features and the PCOS prediction is carried out. All the key phases are explained in the forthcoming sub-sections.

### Data acquisition

**Fig. 1.**
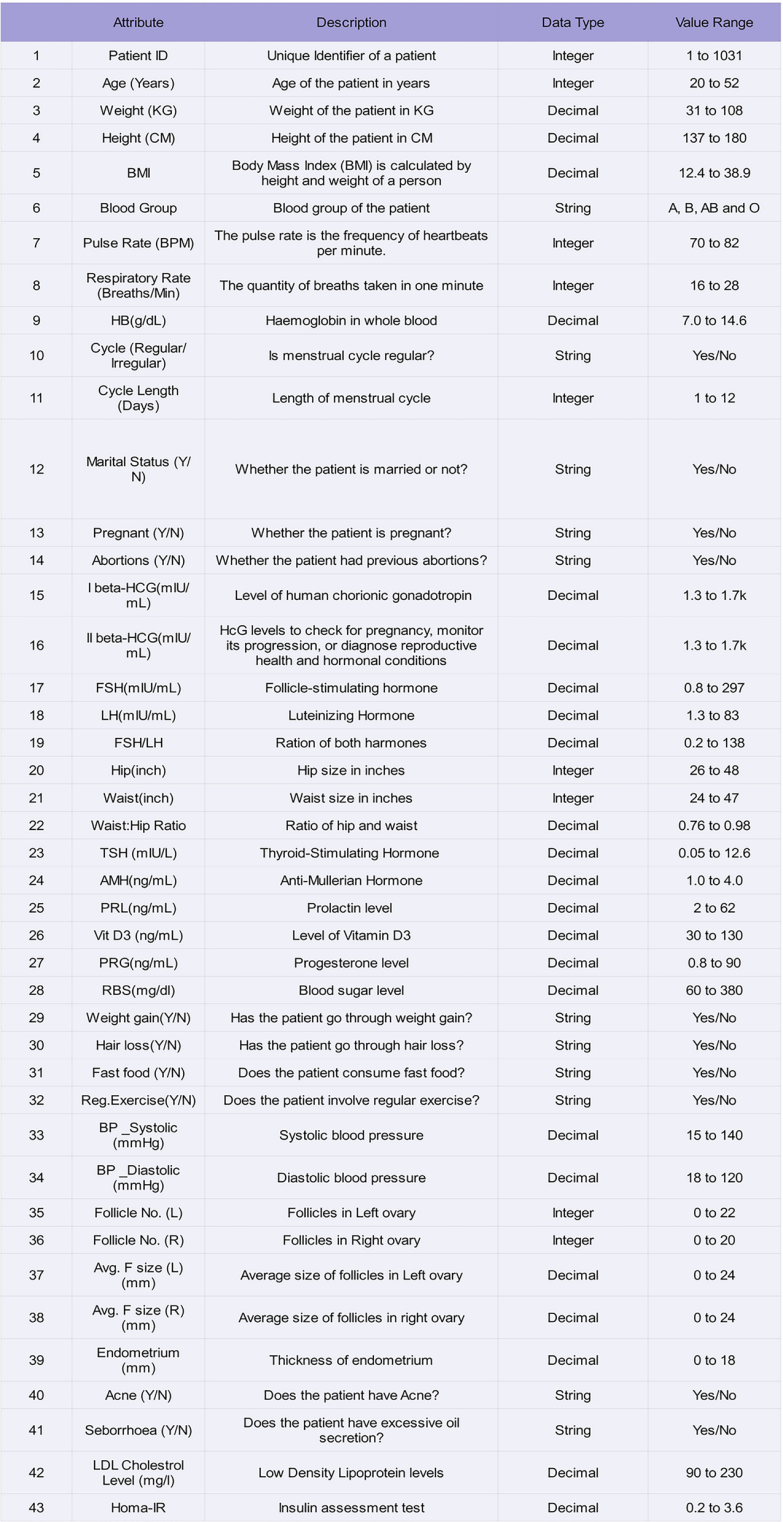
Dataset details.

This work deals with a tailored database being collected from different required data for this research are collected from Kaggle, Mendeleys^29,30^. On analysis, the importance of HOMA-IR is understood and then the dataset is synthesized with the assistance of lab technician and Gynaecologist with 1041 records and the proposed work involves the same attributes as in Kaggle, in addition to Seborrhoea, LDL Cholesterol and Homa-IR (hence, with 43 attributes). The accumulated data are organized and made up with the advice of Lab technicians and Physicians. The raw database contains several attributes, as shown in Fig. 1 The significance of all the attributes is summarized. PCOS exhibits diverse manifestations at various life stages, presenting distinct symptoms and hazards during infancy, adolescent, reproductive years, and postmenopausal periods. Timely identification and intervention can enhance fertility, hormonal equilibrium and overall health in the long run.

Postmenopausal women with PCOS require continuous management for metabolic and cardiovascular disease risk. Consequently, PCOS poses a significant risk, impacting not only reproductive health but also overall well-being. Weight significantly impacts PCOS, affecting hormonal equilibrium, insulin sensitivity, reproductive capabilities, and long-term health hazards. Obesity in PCOS is primarily central (abdominal obesity), resulting in increased visceral fat buildup and metabolic abnormalities. In PCOS, FSH levels are either normal or diminished, resulting in inadequate follicle development and anovulation. In PCOS, increasing LH levels frequently lead to increased androgen synthesis (testosterone, DHEAS). A significant number of women with PCOS exhibit subclinical hypothyroidism, which may exacerbate symptoms. PCOS is characterized by numerous tiny follicles in the ovaries, resulting in elevated levels of Anti-Müllerian Hormone (AMH).

Increased prolactin levels can inhibit ovulation and lead to infertility. Progesterone (PRG) is crucial for the regulation of menstrual cycles and the sustenance of pregnancy. In PCOS, progesterone levels are diminished due to anovulation. In PCOS, women frequently have an elevated Waist-to-Hip Ratio (WHR), signifying central obesity, which is closely associated with IR, metabolic syndrome, and cardiovascular risks. Women with PCOS exhibit an elevated risk of IR, resulting in irregular blood glucose levels and heightened susceptibility to type 2 diabetes and metabolic syndrome.

Women with PCOS experience irregular follicular development, impacting ovulation and fertility. In PCOS, hormonal imbalances, particularly elevated oestrogen, and diminished progesterone, influence endometrial proliferation, desquamation, and fertility. Seborrhoea, characterized by excessive oiliness of the skin and scalp, is a prevalent manifestation of PCOS resulting from elevated testosterone levels that promote increased sebum production. It frequently manifests in conjunction with acne, alopecia, and hirsutism (excessive facial and body hair). Low-Density Lipoprotein (LDL) cholesterol, frequently referred to as "bad cholesterol," is typically high in women diagnosed with PCOS. Elevated LDL levels heighten the risk of cardiovascular disease, cerebrovascular accidents, and metabolic syndrome.

HOMA-IR (Homeostatic Model Assessment for IR) is a crucial assessment for quantifying IR, a significant contributor to PCOS. IR impacts around 70% of women with PCOS, elevating the risk of type 2 diabetes, obesity, infertility, and cardiovascular disease. HOMA-IR evaluates insulin efficacy in the body and informs treatment strategies. Vitamin D3 (cholecalciferol) is essential for the regulation of hormones, insulin sensitivity, and inflammation. Between 67 and 85% of women with PCOS exhibit Vitamin D insufficiency, exacerbating IR, hormonal imbalance, and reproductive complications. In case of string datatype, 1 indicates ‘Yes’ and 0 indicates ‘N’. The total PCOS positive and negative samples in the dataset are 356 and 684 respectively.

**Fig. 2:**
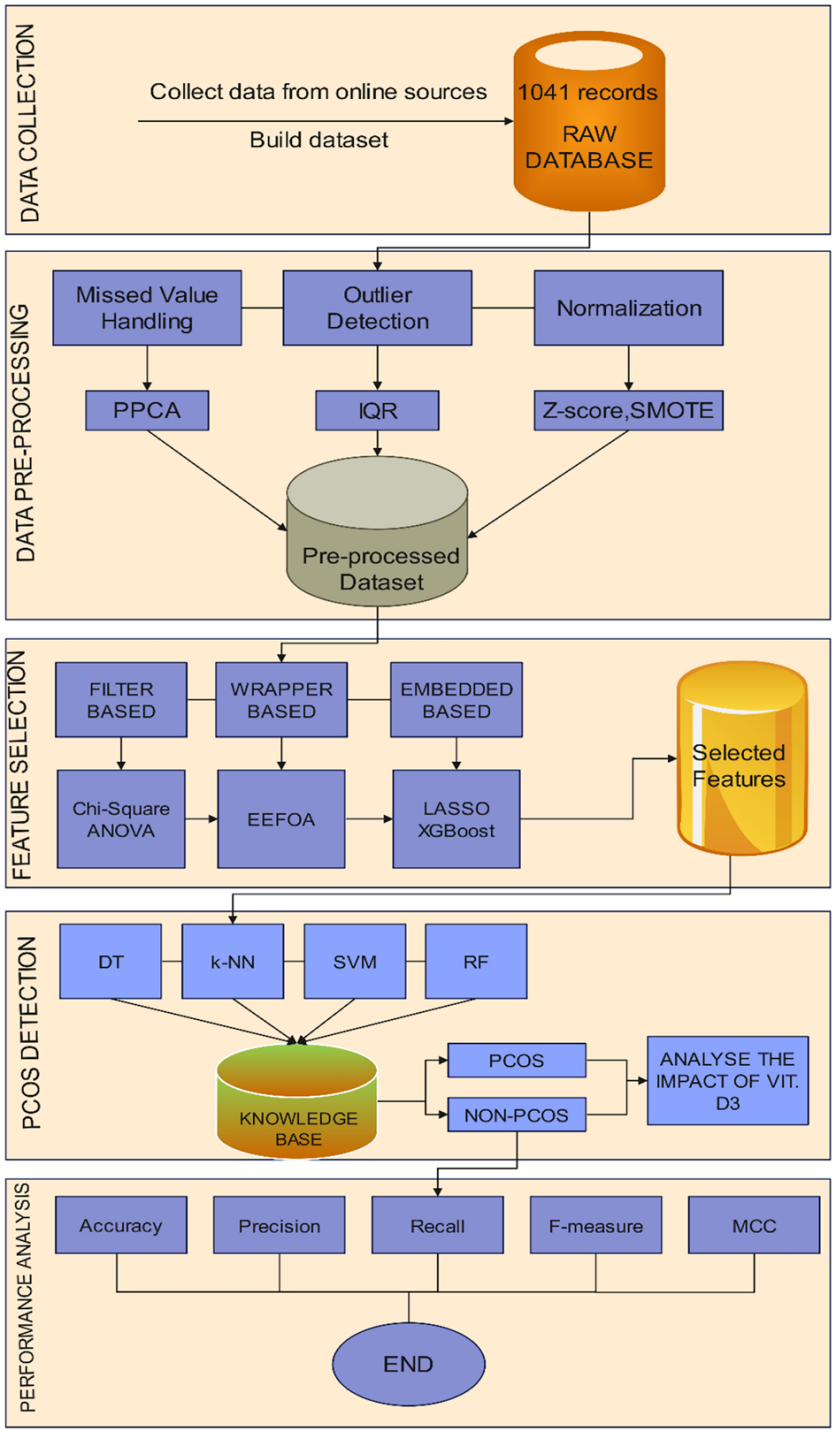
Overall block diagram of the proposed work.

Due to the important implications, the proposed work has collected all these attributes for predicting PCOS and analysing the impact of vitamin D with it. Once these attributes are acquired, data pre-processing is carried out. The overall flow of the proposed work is shown in Fig. 2.

#### Data pre-processing

When processing medical datasets, it is highly crucial to pre-process the acquired data, such that the classification accuracy can be enhanced. Poor data pre-processing seriously influences the overall efficiency of the ML algorithm. This work pre-processes the acquired data by considering three perspectives such as missed value handling, outlier detection and data standardization, which are handled by Probabilistic Principal Component Analysis (PPCA) and Interquartile Range (IQR) respectively. All the features should be comparable and hence data standardization is necessary, for which Z-score standardization is employed.

#### Missed value handling

PPCA utilizes the Expectation–Maximization (EM) technique to repeatedly compute the Maximum Likelihood Estimates (MLE) for any given incomplete dataset. This technique performs dimensionality reduction by reducing the reconstruction error rates and variance loss at data compression by minimizing the Euclidean distance from original to projected data points. Various PCA techniques for addressing missing data have been presented^31,32^, differing in their assumptions regarding the links between the original and the latent data points. MLE is used to evaluate the unknown or missing parameters of data^33^. EM algorithm involves two phases such as the Expectation (E) step and the Maximization (M) step^34^ in every iteration. Step ‘E’ considers the observed values and estimates all missing values in the dataset^34,35^. Step ‘M’ phase subsequently updates the imputed parameters by MLE, by employing the whole dataset generated in the step ‘E’. This iterative procedure, comprising both "E&M" processes, persists until no additional enhancements can be made to the likelihood estimates^35^. This procedure yields imputed datasets with MLE indicating the enhanced precision of the imputed values.

#### Outlier detection by IQR

IQR is employed to detect anomalies, which are values that are either above the 75th percentile or below the 25th percentile by 1.5 times the IQR. It should be decided either to limit these outliers to the boundary values or to eliminate them. This outlier detection technique works well for dataset with mixed datatypes.

#### Data balancing by Z-Score standardization and SMOTE

The Z-score normalization procedure is employed to standardize the features, to make them fall under a comparable scale. This process helps in achieving better training performance and interpretability. The Z-score is computed by the following equation.

1 $${Z}_{Score}=\frac{DS-\mu }{\sigma }$$

In the above equation, $DS, \mu$ and $\sigma$ indicate data sample, mean and standard deviation respectively.

**Fig. 3:**
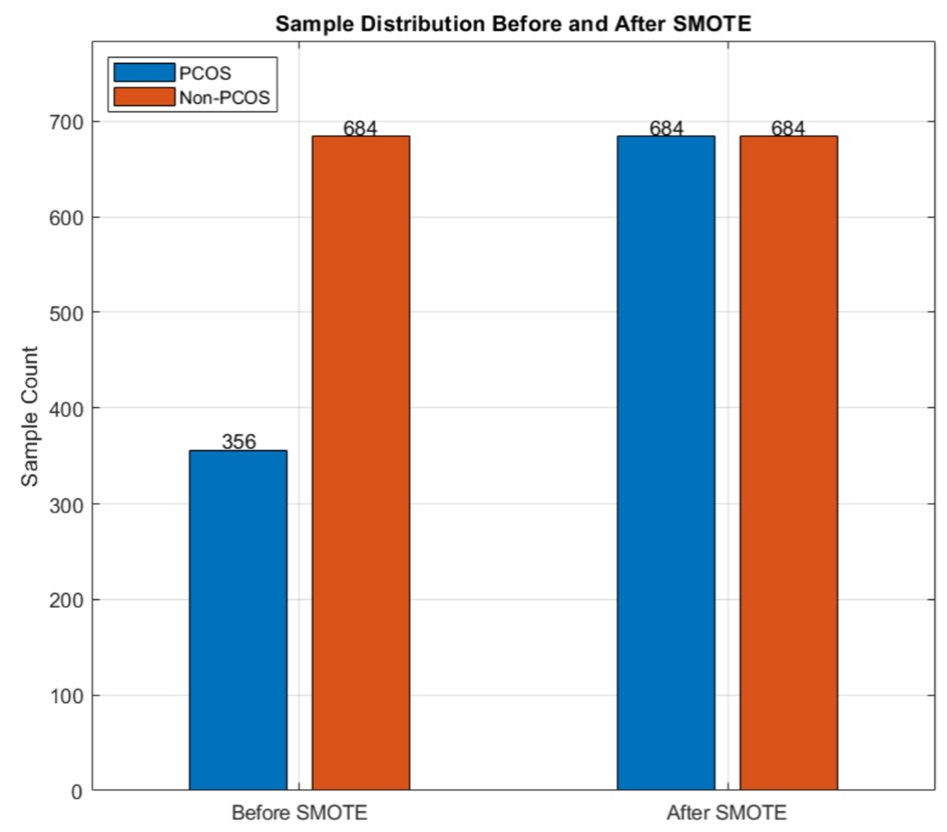
Sample distribution before and after SMOTE.

Synthetic Minority Over-sample Technique (SMOTE) is employed for balancing the class distribution of a dataset. Initially, the total PCOS positive and negative samples in the dataset are 356 and 684 respectively. As the class imbalance is evident, the prediction may end up with the prediction of class with majority samples. This kind of biased classification is prevented by SMOTE and the samples are balanced as 684 in each class respectively. The total samples are improved to 1368 after the application of SMOTE, as depicted in Fig. 3. Initially, the PCOS and Non-PCOS cases were 356 and 684 records, which was applied SMOTE for avoiding biased prediction. Hence, the positive samples are increased with the total samples of 1368, as shown in Fig. 3.

Hence, the data pre-processing of this work attempted to handle missed values, remove outliers and standardize the dataset. The pre-processed dataset is then dealt for feature selection, which is described as follows.

### Significant feature selection by exploration of different methods

Feature selection is crucial for enhancing the performance of a ML model, as significant features alone are enough to train a model, rather than the utilization of all available features. The feature selection approach helps in reducing the computational complexity, as well. Numerous features such as hormonal, lifestyle, metabolic attributes are available in the dataset. However, it is recommended to process the relevant features that could make the classification easier. The issue of overfitting can also be avoided by handling relevant features. Understanding the significance, this work explores three different feature selection methods such as filter, wrapper and embedded methods.

#### Filter based feature selection approach

In this category of feature selection approach, statistical approaches such as Chi-square test (${\chi }^{2}$) and Analysis of Variance (ANOVA) are employed. Both these tests are meant for selecting the feasible features based on their correlation with each other.

${ \chi }^{2}$-It works better for categorical attributes, while ANOVA performs better for numerical attributes. ${\chi }^{2}$ measures the degree of independence between the feature $f{t}_{i}$ and class label $c{l}_{i}$, while the comparison is made by chi distribution with the degree of independence set to 1.

2 $${\chi }^{2}\left(f{t}_{i},c{l}_{j}\right)=\frac{N.{\left(FD-CLDW\right)}^{2}}{(F+DW)(F+D)(DW+D)(CL+D)}$$

In the above equation, $N$ is the total count of records in the dataset, $F$ denotes the feature frequency of $f{t}_{i}$ and class label $c{l}_{j}$. $D$ is the frequency that does not hold $f{t}_{i}$ and $c{l}_{j}$ together in the dataset, whereas $CL$ is the occurrence frequency of $c{l}_{j}$ without $f{t}_{i}$ and $DW$ indicates the occurrence frequency of $f{t}_{i}$ without $c{l}_{j}$. The value of $i$ and $j$ ranges between (1 and 43) and (0,1) respectively.

**Fig. 4:**
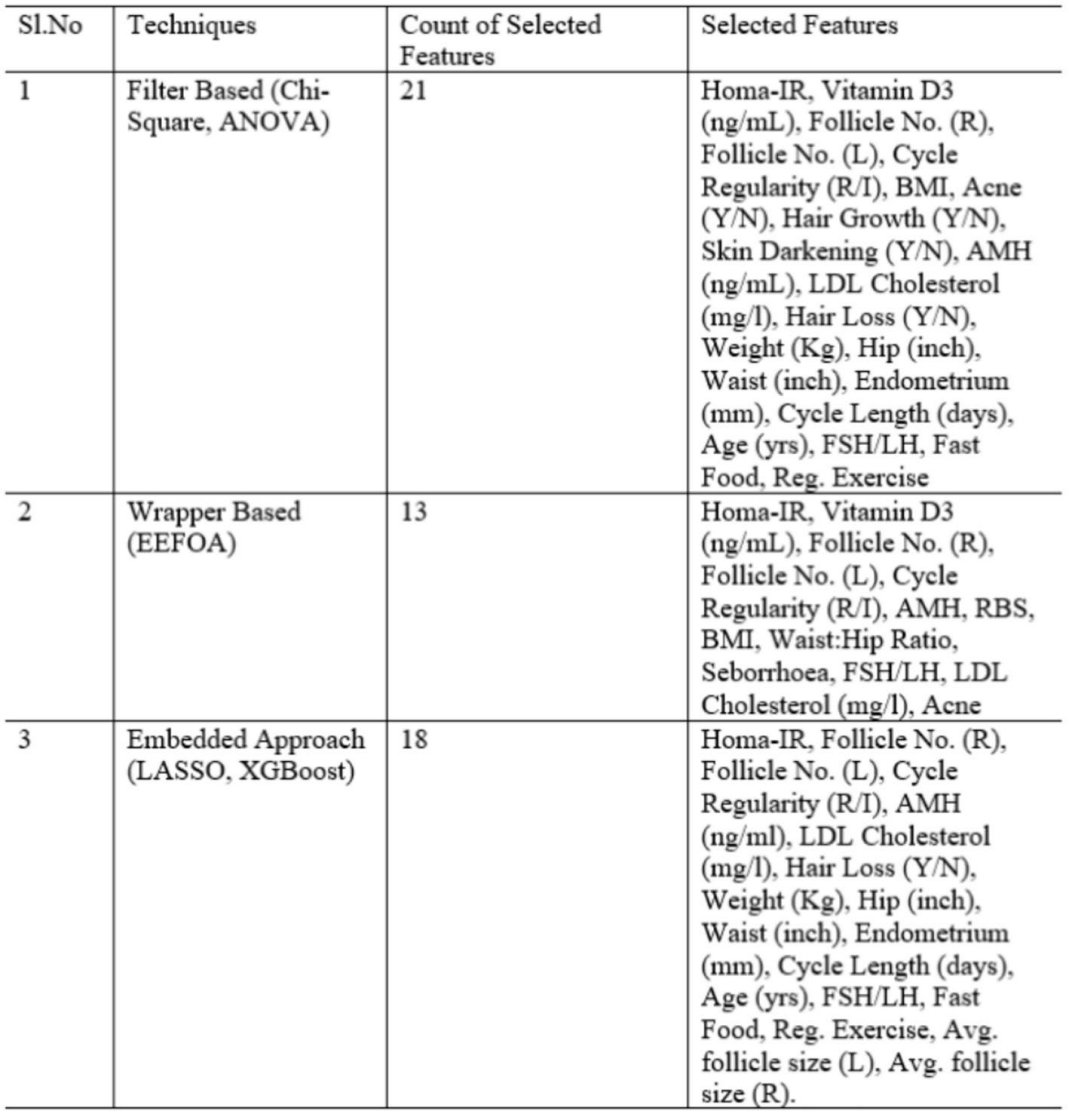
Feature selection by different techniques.

ANOVA - This is a statistical approach, which compares the mean of attributes for different classes. Hence, it paves way for selecting the appropriate features that can effectively classify between classes. Variance Between (VBG) and Within (VWG) Groups are considered by this approach and greater VBG than VWG indicates the significance of the feature during classification. The features selected by both these techniques are listed in Fig. 4.

Hence, the ${\chi }^{2}$ and ANOVA scores are obtained to select the vital features, which relies on statistical scores. On the positive side, these techniques are independent and do not rely on ML model, which makes it to be faster and computationally inexpensive. On the negative side, feature interactions are not considered.

#### Wrapper based feature selection approach

This method of feature selection is so powerful, that it can evaluate varied feature subsets by employing a ML model, while ensuring accuracy and robustness of feature selection in performing a classification task. This approach ensures to produce the best choice of features from a pool of features. Though the process is bit time consuming, the feature selection is optimal and hence, the overall accuracy of classification is enhanced.

##### Electric eel foraging optimization algorithm (EEFOA)

Under the wrapper category-based feature selection, this work employs EEFOA^36^. Numerous bio-inspired algorithms are explored in the existing literature for medical applications^37–41^. The EEFO is a bio-inspired metaheuristic algorithm that emulates the foraging behaviour of electric eels based on their electric field interactions. The method equilibrates exploration (seeking solutions) and exploitation (enhancing optimal solutions) to identify the ideal feature subset. The major steps involved in this algorithm are initialization, interaction of electric field, foraging movement, exploration, exploitation and convergence.

Initially, the electric eel population is generated and every eel emits an electric field with respect to its fitness. The eels can then move upon to high energy fields for better exploration. Certain eels reach near the optimal solution and the rest explore the fields. Whenever the most optimal solution or the maximum iteration is reached, the algorithm converges. The reasons for the choice of EEFOA are its ability to handle complex data, balanced exploration and exploitation to avoid local optima.

The initialization of control parameters, including the maximum number of iterations and the size of the electric eel population, is the first step in the EEFO process. A random set of eels is generated, and each eel exhibits interactive behaviour during exploration when the energy factor (E) exceeds 1. When the energy factor (E) is less than or equal to 1, each eel engages in exploitation with the same probability during resting, migrating, or hunting. The most effective solution is iteratively updated after candidate solutions are generated and compared to existing ones. The energy factor (E) decreases as the iteration advances, resulting in a transition from exploration to exploitation. The interactive process continues until a predetermined halting condition is achieved, at which point the optimal solution is retained.

Eels, capable of detecting prey locations via mild electric discharges, adeptly modify their postures. During foraging, if an eel detects the proximity of prey, it transitions to a potential location; if not, it remains stationary. The fitness of the eel is computed by the following equation.

3 $${X}_{i}\left(t+1\right)=\left\{\begin{array}{c}{X}_{i}\left(t\right) fit\left({X}_{i}\left(t\right)\right)\le fit\left({v}_{i}\left(t+1\right)\right)\\ {v}_{i}\left(t+1\right) fit\left({X}_{i}\left(t\right)\right)>fit\left({v}_{i}\left(t+1\right)\right)\end{array}\right.$$

where, $fit\left({X}_{i}\left(t\right)\right)$ denotes the fitness of ${i}^{th}$ eel in its candidate position, ${X}_{i}$ and ${v}_{i}$ are the positions of a random eel and randomly chosen food respectively. In case of this work, the fitness function is the ANOVA scores. The pseudocode of EEFOA is presented as follows.


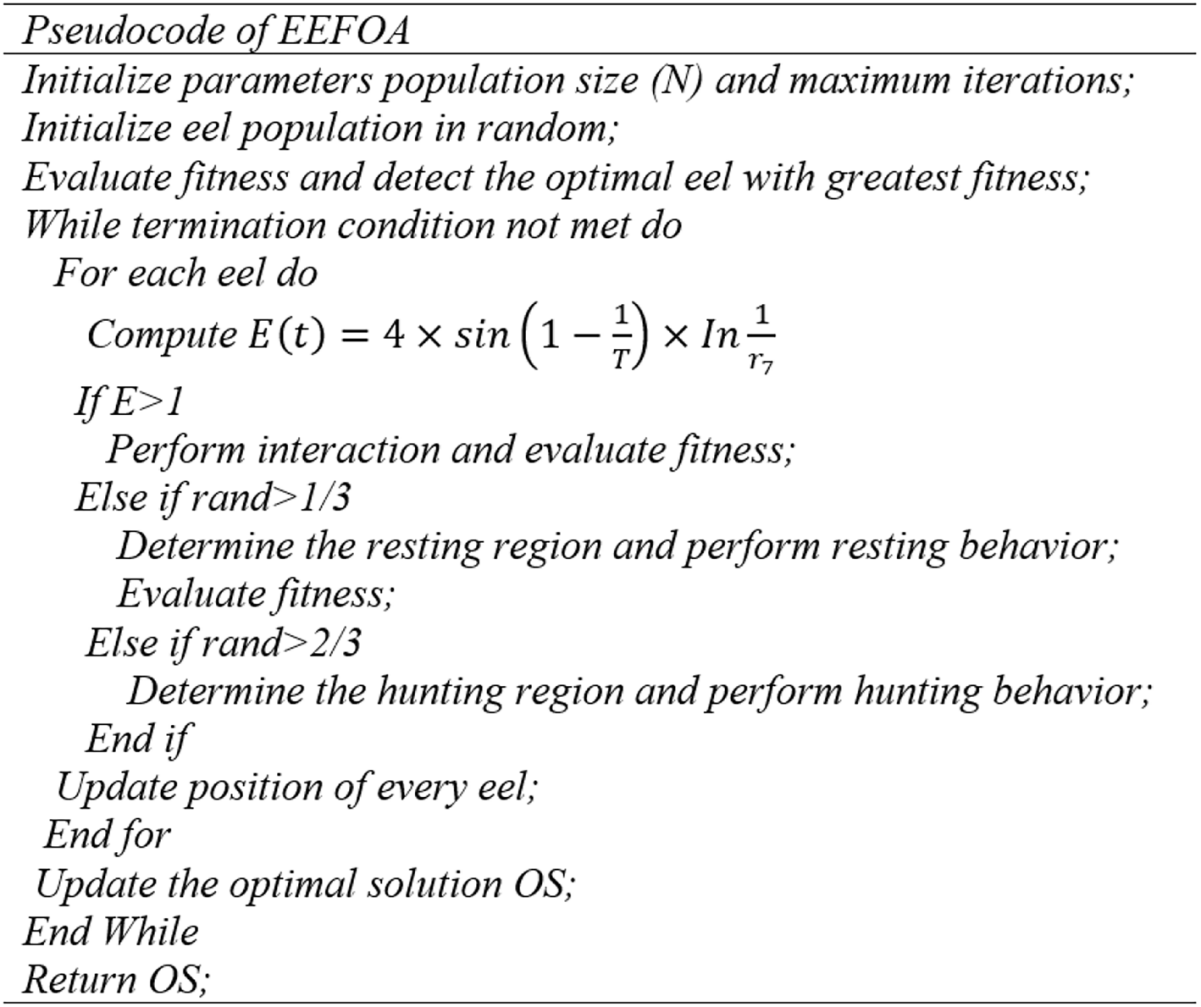


Each eel utilizes its interactive behaviour to engage in investigation during each iteration, provided that the energy factor E is greater than 1. Conversely, when the energy factor E is equal to or less than 1, each eel has an equal likelihood of participating in exploitation, regardless of whether it is resting, migrating, or foraging. Each case is applied to each eel to generate new candidate solutions, which are then compared to existing ones. Throughout the iteration process, the most current and optimal solution is consistently updated. The energy factor E decreases as the iteration advances, causing each eel to transition from exploration to exploitation. This interactive process persists until the specified termination condition is satisfied. The most effective solution that has been attained to that point is then retained.

On analysis, it is observed that the count of features selected by filter and wrapper methods differ with a good range. However, the efficiency of feature selection can be determined by the classification accuracy of the ML model.

#### Embedded method based feature selection

This kind of feature selection executes during the process of model training and hence, the features are automatically selected during the training process of ML model. The features are given scores during the training process and the features with more importance are retained, while the irrelevant features are eliminated. The embedded techniques can handle voluminous datasets and avoids overfitting.

- LASSO—This work employs Least Absolute Shrinkage and Selection Operator (LASSO) regression and XGBoost under this category. The LASSO regression is based on L1 penalty and the insignificant features are set to 0 and is computed by the following equation.

4 $${\text{min}\left|\left|Y-XW\right|\right|}^{2}+\lambda {\left|\left|W\right|\right|}_{1}$$

Here, $\lambda$ is the regularization parameter and ${\left|\left|W\right|\right|}_{1}$ is the sum of absolute weights.

- XGBoost – It is a gradient boosting DT based on regularized learning meant for ensemble learning. The II order Taylor series of loss function ($L$) at ${t}_{o}-{t}_{h}$ iteration is given by

5 $${L}^{t}\cong \sum_{i=1}^{k}\left[l\left({y}_{i},{y}_{i}^{t-1}\right)+g{r}_{i}{f}_{t}\left({x}_{i}\right)+\frac{1}{2}sg{r}_{i}{f}_{t}^{2}\left(x\right)\right]+\Omega ({f}_{t})$$

- In the above equation, ${f}_{t}$ represents ${t}_{o}-{t}_{h}$ tree, $g{r}_{i}$ and $sg{r}_{i}$ indicate the gradients of I and II order respectively. Here, the optimal split node is determined by using gain (GN), while training XGBoost, as shown in Eq. (6).
  6 $$GN=\frac{1}{2}\left[\frac{{\left(\sum_{i\in {I}_{L}}g{r}_{i}\right)}^{2}}{\sum_{i\in {I}_{L}}sg{r}_{i}+\lambda }+\frac{{\left(\sum_{i\in {I}_{R}}g{r}_{i}\right)}^{2}}{\sum_{i\in {I}_{R}}sg{r}_{i}+\lambda }-\frac{{\left(\sum_{i\in I}g{r}_{i}\right)}^{2}}{\sum_{i\in I}sg{r}_{i}+\lambda }\right]-\gamma$$

- The segmented samples of left and right nodes are denoted by ${I}_{L}$ and ${I}_{R}$; ${I=I}_{L}\cup {I}_{R}$. The penalty parameters are $\lambda$ and $\gamma$. The gain score for every tree split is indicated by gain and the feature significance score is computed by average gain. The average gain is calculated by dividing the total gain of all trees by the total number of divisions for each feature. The significance of the corresponding feature is directly proportional to feature importance score of XGBoost.

Hence, the feature selection process of this work explored three categories by considering filter, wrapper and embedded approaches. The selected features are employed to train the ML models, as described below.

#### PCOS classification by ML models

ML approaches employed by this work to classify between PCOS affected and unaffected classes are DT, SVM, RF and k-NN. The overall algorithm of the proposed work is presented below.
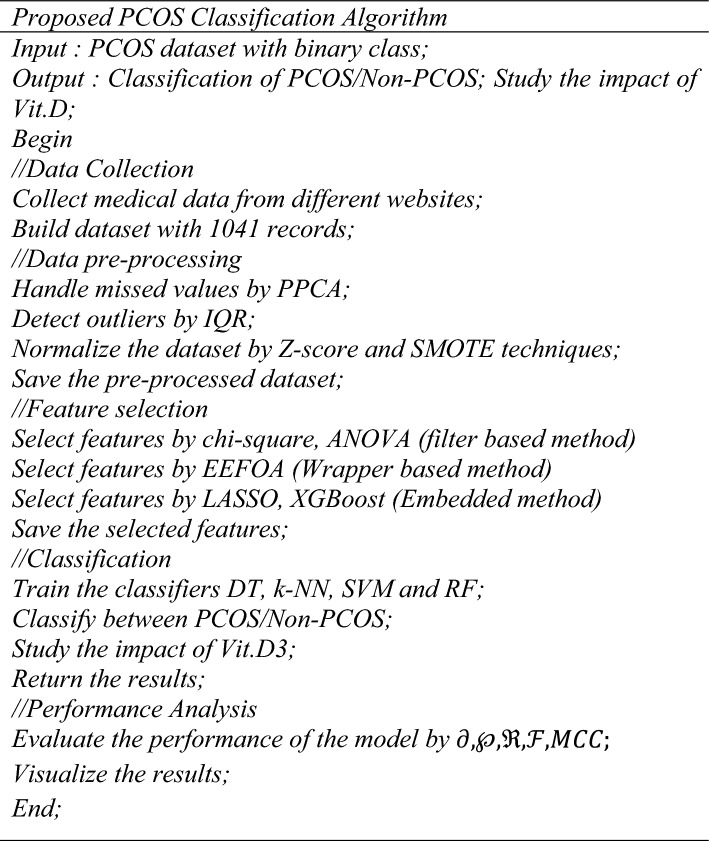


#### DT

DT is one of the popular and promising classification models, owing to its simplicity and straightforwardness. The DT encompasses leaves and branches, where leaves denotes class and branches are denoted by the test results. Usually, the learning process of DT involves upside-down processing and root node is the base from where the best feature is selected. The operation of tree split-up is carried out then and there. While grouping, every feature is compared with the threshold value in every test. The DT works well for all datatypes and hence, it is suitable for all sorts of classification and regression problems. The impurity of dataset is measured by entropy, which lies in between 0 and 1, where 0 is preferrable and 1 is not. The entropy is computed by

7 $$Ent\left(s\right)=\sum_{i=1}^{C}{P}_{i}\text{log}{2}^{{P}_{i}}$$

Here, $C$ is the number of classes, ${P}_{i}$ is the subset’s sample number divided by the ${i}^{th}$ attribute. The subset is a value that can be held by a feature. Information Gain is another variable that determines the scope of variable. The greater the value, the better is the performance.

8 $$Gain\left(S\right)=Ent\left( Y\right)-Ent(X,Y)$$

Though, DT offers several advantages such as simplicity and computational efficiency, it suffers from the issue of overfitting and the consistency of the tree depends on the stability of the dataset.

#### RF Classifier

RF classifier is an ensemble-based classification algorithm, as it is based on several DTs. The outcomes of all the DTs are considered to arrive at a single outcome, such that the accuracy of decision making is enhanced^42^. In place of individual decision, RF employs multiple learning algorithms to arrive at a decision, at the cost of computational complexity, yet with better accuracy rates. This classifier generates and aggregate several trees and let the data and decisions be denoted by $Dt=\{{dt}_{1},{dt}_{2},{dt}_{3},\dots ,{dt}_{n}\}$ and $Dn=\{d{n}_{1}, d{n}_{2},d{n}_{3},\dots ,d{n}_{n}\}$. The lower and upper bound are represented by $b=1$ and $UB=B$.

The data sample $ds{\prime}$ is classified by computing the mean classification ($MC$) from multiple trees. The individual decision made by the tree is given by

9 $$ID=\sum_{b=1}^{B}{f}_{b}(d{s}{\prime})$$

10 $$MC=\frac{1}{B}\sum_{b=1}^{B}{f}_{b}(d{s}{\prime})$$

The RF is insensitive to noise, supports parallel processing works well with missing data. However, RF is computationally expensive and consumes time to process.

#### k-NN

The k-NN classifier is a supervised classifier that acquires knowledge from the training data and is capable of distinguishing between PCOS affected and unaffected classes^43^. The Euclidean distance between the trained dataset and the data that was obtained is calculated by the classifier, as shown in Eq. (11).

11 $${E}_{D}=\sum_{i=1}^{N}\sqrt{{u}_{i}^{2}-{v}_{i}^{2}}$$

The quality of classification is contingent upon the selection of ‘k’ and it is challenging to detect the optimal value of ‘k’. Therefore, this study implements k-fold cross validation, which independently determines the k value. The training data is divided into k samples, and a single sample is designated as the test entity. The remaining entities are treated as the training samples. This process is repeated k times, each time altering the test sample until all samples have been selected as the test sample. Ultimately, the mean value of the k results is determined and designated as k. The k-NN classifier can differentiate between the two classes in this manner.

#### SVM classifier

The chosen features and the calculated threshold are used to train the supervised classification algorithm SVM^44^. Assume that {1, 2, 3,…N} samples must be categorized as either unaffected or impacted by PCOS. A hyperplane is used to divide the two classes; the hyperplane must be carefully chosen because it affects the classification accuracy. The following equation divides the hyperplane.

12 $$f\left(x\right)=\sum_{i=1}^{N}{\beta }_{i}{\psi }_{i}\left(c{l}_{1},c{l}_{2}\right)+th$$

The hyperplane of the classifying region ${\psi }_{i}\left(c{l}_{1},c{l}_{2}\right)$. is separated by the lagrange multiplier ${\beta }_{i}$ in the equation above. $th$ stands for the threshold that is being used to distinguish between the two classes. The trial-and-error method is used to choose the threshold.

Hence, four different classifiers are trained with the selected features and the Explainable AI (XAI) techniques are discussed in the following section.

### XAI to emphasize feature importance

**Fig. 5:**
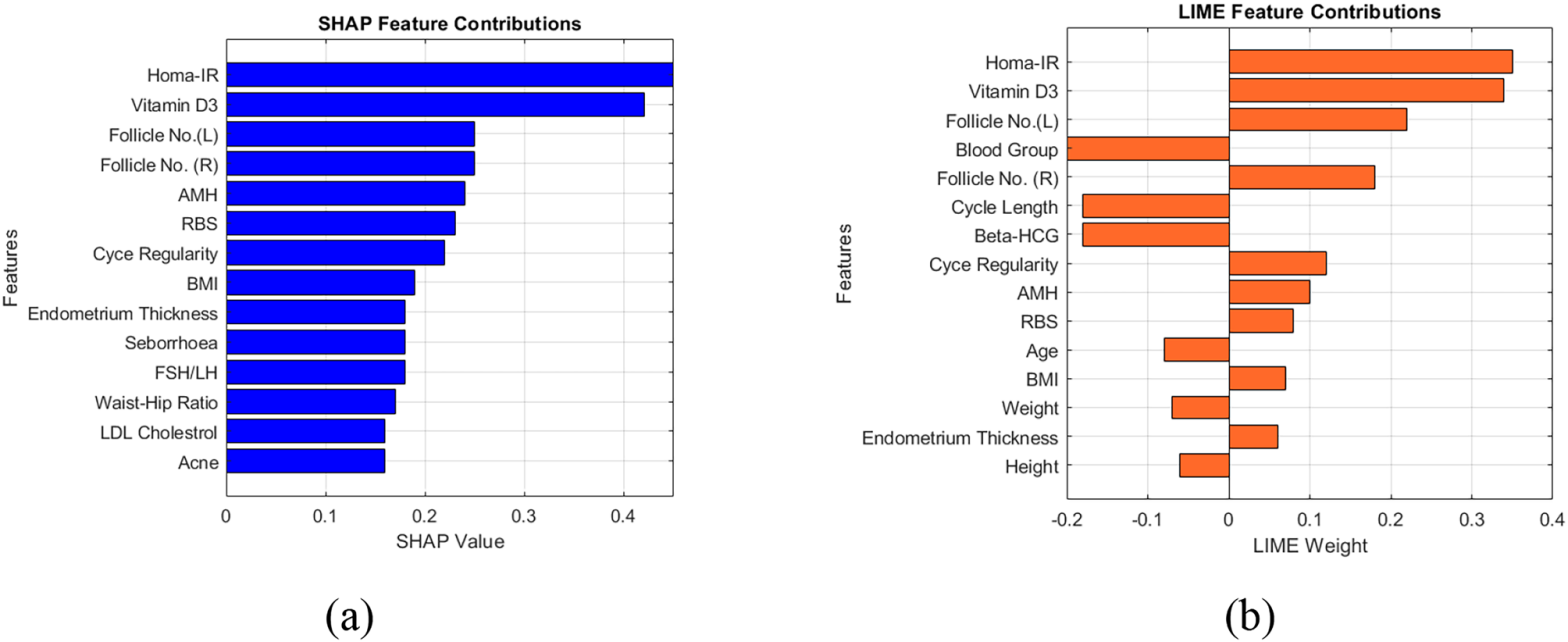
XAI interpretations (**a**) SHAP (**b**) LIME.

The inclusion of XAI in any ML model brings in different merits such as easy interpretability, better transparency and understandability. Most popular XAI methods are Shapley Additive Explanations (SHAP) and Local Interpretable Model Agnostic Explanations (LIME). The importance of every feature is interpreted by these models; however, they have nothing to do with performance evaluation. The SHAP values highlights the importance of a specific feature in predicting the class of any given sample. Similarly, LIME is also a result interpretation tool for ML models. This work employs RF classifier and the illustrations are presented in Fig. 5.

Each bar indicates the feature contribution towards the final prediction. The features at the top and bottom denote the most and least influential features for final prediction. As per the interpretation, HOMA-IR is a critical marker that shows the role of insulin resistance in PCOS, which means glucose metabolism screening is essential for PCOS prediction. Vitamin D3 is a highly relevant feature, where low levels may relate to PCOS. Follicle counts and AMH do correlate with ultrasound based diagnostic criteria that validates the consistency of the model. Hormonal, metabolic and reproductive features such as menstrual cycle irregularity, BMI and RBS are also considered significant. Acne, LDL, waist-hip ratio are all still relevant but minimally predictive for this model. Hence, the top predictors of this model are Homa-IR and vitamin D3 deficiency. From the interpretations, it can be noted with clarity that HOMA-IR and Vitamin D3 are the most important features in discriminating the PCOS affected and unaffected classes. The Blood group, cycle length, Beta-HCG are found to be insignificant in classification. The performance analysis of the work is discussed in the following section.

## Results and discussion

The proposed work is implemented in Matlab2021A on a stand-alone computer with 16 GB RAM. The train/test split up is 80/20, such that 1095 images are trained and 273 images are tested. The performance of the proposed work is tested in different perspectives and the following sub-sections discuss the performance measures along with the attained results.

### Performance measures

Conventional performance indicators are employed to evaluate the efficacy of the work. The measures encompass accuracy (∂), precision (℘), recall ($\mathfrak{R}$), F-score ($\mathcal{F}$), and Matthews Correlation Coefficient (MCC). The efficacy of the work is assessed by utilizing Eqs. (13–17). ∂ represents the proportion of correctly classified samples relative to the total number of samples. ℘ denotes the ratio of accurately predicted positive cases relative to the total number of anticipated positive samples

13 $$\partial =\frac{{T}_{Pos}+{T}_{Neg}}{{T}_{Pos}+{T}_{Neg}+{F}_{Pos}+{F}_{Neg}}$$

14 $$\wp =\frac{{T}_{Pos}}{{T}_{Pos}+{F}_{Pos}}$$

15 $$\mathfrak{R}=\frac{{T}_{Pos}}{{T}_{Pos}+{F}_{Neg}}$$

16 $$\mathcal{F}=2\times \frac{(\mathfrak{\wp }\times \mathfrak{R})}{(\mathfrak{\wp }+\mathfrak{R})}$$

17 $$MCC=\frac{\left({T}_{Pos}\times {T}_{Neg}\right)-({F}_{Pos}\times {F}_{Neg})}{\sqrt{({T}_{Pos}+{F}_{Pos})({T}_{Pos}+{F}_{Neg})({T}_{Neg}+{F}_{Pos})({T}_{Neg}+{F}_{Neg})}}$$

$\mathfrak{R}$, commonly known as sensitivity or true positive rate, indicates the ratio of correctly identified positive samples to the total number of actual positive samples. The value of $\mathcal{F}$ is ascertained by calculating the harmonic mean of ℘ and $\mathfrak{R}$. The Matthews Correlation Coefficient (MCC) is a statistical metric utilized to measure the connection between observations and predictions. Considering both true and false positives, as well as negatives, it produces a result that can vary from -1 to + 1.

### Performance analysis w.r.t feature selection techniques and classifiers

Feature selection is the most significant process that decides the efficiency of the ML model. As this work entails 43 features, it is necessary to weed out irrelevant features from the dataset, to minimize computational, space and time overheads. Selection of relevant features helps in attaining better classification accuracy rates, however on the other hand, when the feature selection technique does not suit the dataset, then the classification accuracy rates can be affected. Hence, the choice of feature selection technique is given utmost importance and this work explores different methods of feature selection such as filter based (${\chi }^{2}$, ANOVA) wrapper based (EEFOA) and embedded techniques (LASSO, XGBoost). The filter approaches involve statistical analysis for selecting features, while the wrapper-based method select features intelligently. The embedded techniques select features on-the-go and hence, supports scalability and generalization.

On analysis, this work detects that wrapper-based technique performs well for the incorporated PCOS dataset and intelligent selection of features works better. The parameters utilized for selecting features are tabulated in Table 1.

**Table 1:** Hyperparameters with values.

Parameters	Values
Size of population	30
Iteration count	500
Energy decay rate	0.95
Initial energy level	2
Foraging probability	0.2
$\alpha ,\beta$	1.5,0.5

**Fig. 6:**
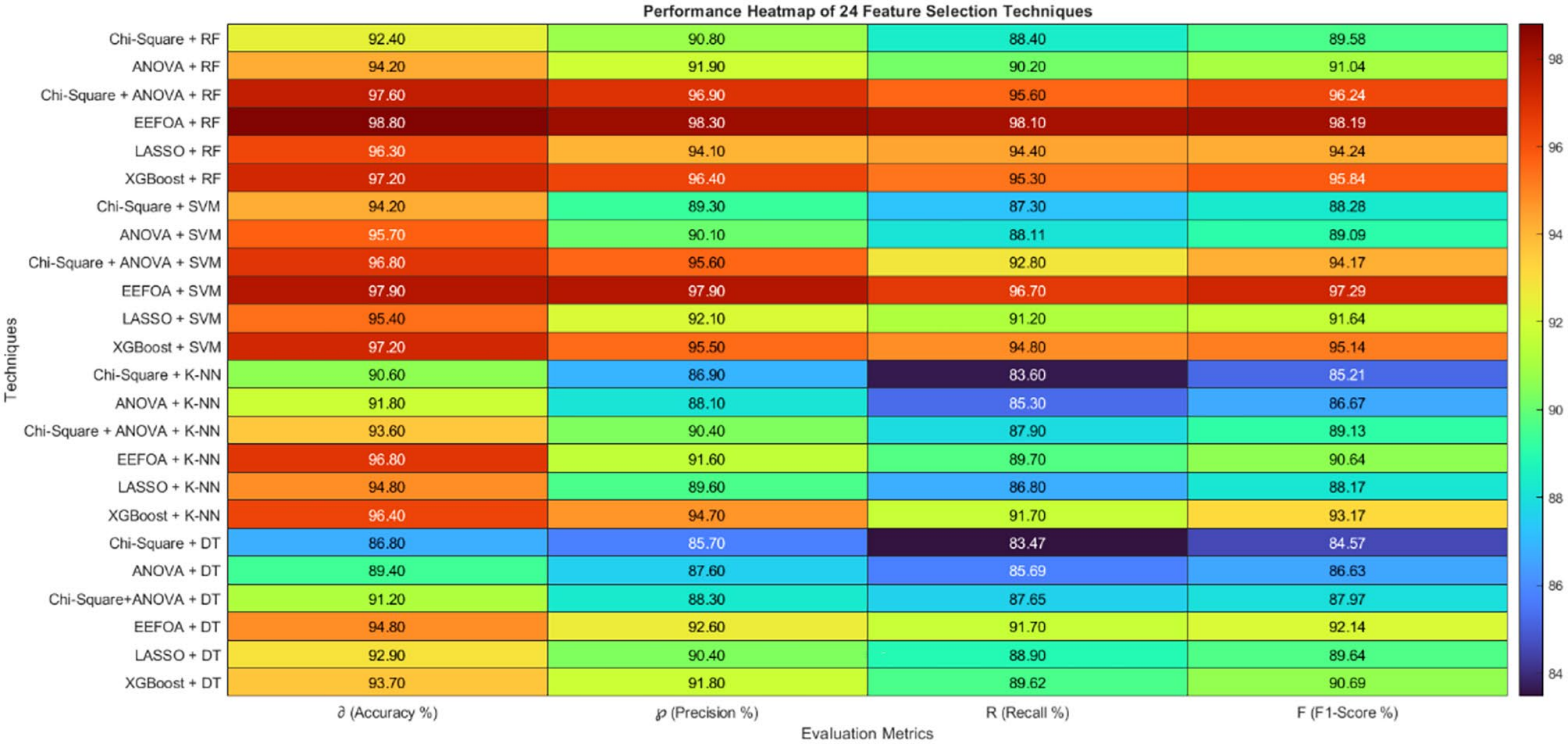
Performance heatmap of different feature selection techniques and classifiers.

The analysis is carried out by varying the feature selection techniques, in addition to the classifier. The classifiers being explored by this work are DT, k-NN, SVM and RF. The attained results are shown in Table 2 and its heatmap in Fig. 6.

**Table 2:** Comparison analysis by varying feature selection techniques and classifiers.

Techniques	$\partial$(%)	$\wp$(%)	$\mathfrak{R}$(%)	$\mathcal{F}$(%)
DT without feature selection	78.3	76.4	72.7	74.5
k-NN without feature selection	80.2	79.3	74.6	76.87
SVM without feature selection	83.4	81.7	76.1	78.8
RF without feature selection	86.3	83.4	81.8	82.59
Chi-Square + RF	92.4	90.8	88.4	89.58
ANOVA + RF	94.2	91.9	90.2	91.04
Chi-Square + ANOVA + RF	97.6	96.9	95.6	96.24
**EEFOA + RF**	**98.8**	**98.3**	**98.1**	**98.19**
LASSO + RF	96.3	94.1	94.4	94.24
XGBoost + RF	97.2	96.4	95.3	95.84
Chi-Square + SVM	94.2	89.3	87.3	88.28
ANOVA + SVM	95.7	90.1	88.11	89.09
Chi-Square + ANOVA + SVM	96.8	95.6	92.8	94.17
EEFOA + SVM	97.9	97.9	96.7	97.29
LASSO + SVM	95.4	92.1	91.2	91.64
XGBoost + SVM	97.2	95.5	94.8	95.14
Chi-Square + K-NN	90.6	86.9	83.6	85.21
ANOVA + K-NN	91.8	88.1	85.3	86.67
Chi-Square + ANOVA + K-NN	93.6	90.4	87.9	89.13
EEFOA + K-NN	96.8	91.6	89.7	90.64
LASSO + K-NN	94.8	89.6	86.8	88.17
XGBoost + K-NN	96.4	94.7	91.7	93.17
Chi-Square + DT	86.8	85.7	83.47	84.57
ANOVA + DT	89.4	87.6	85.69	86.63
Chi-Square + ANOVA + DT	91.2	88.3	87.65	87.97
EEFOA + DT	94.8	92.6	91.7	92.14
LASSO + DT	92.9	90.4	88.9	89.64
XGBoost + DT	93.7	91.8	89.62	90.69

**Fig. 7:**
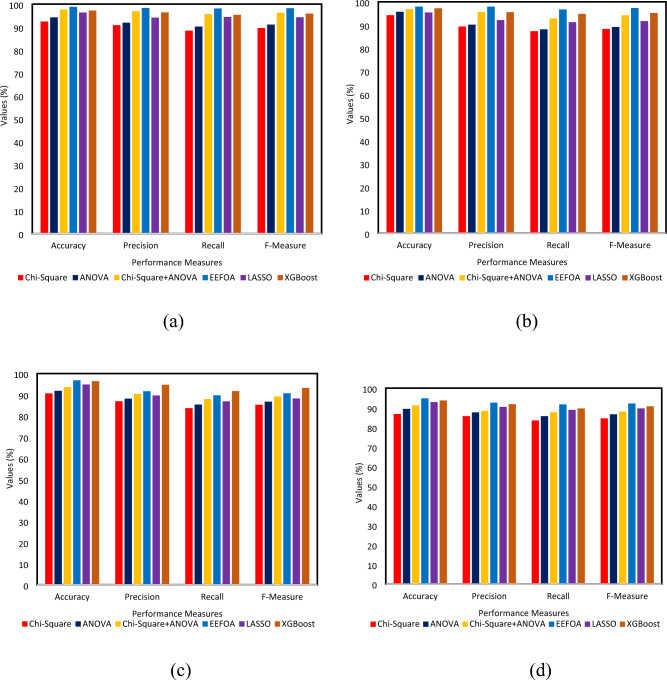
Performance comparison of different feature selection techniques with (**a**) RF (**b**) SVM (**c**) k-NN (**d**) DT.

The analysis is carried out by varying the feature selection techniques such as chi-square, ANOVA, chi-square + ANOVA, EEFOA and XGBoost upon different classifiers such as DT, SVM, RF and k-NN. The experimental results show that the performance of wrapper based feature selection (EEFOA) in association with RF performs well with an $\partial$ rates of 98.8%, the $\wp ,\mathfrak{R}$ and $\mathcal{F}$ rates of 98.3%, 98.1% and 98.19% respectively. The graphical representation of the attained results is shown in Fig. 7.

The second-best performer that follows the previously stated one is EEFOA with SVM by proving $\partial$, $\wp ,\mathfrak{R}$ and $\mathcal{F}$ rates 97.9%, 97.9%, 96.7% and 97.29% respectively. Chi-square + ANOVA features in combination with RF performs well with an $\mathcal{F}$ rate of 96.24% and when the features are employed with SVM, the $\mathcal{F}$ rate is 94.17%. The XGBoost features with RF and SVM prove $\mathcal{F}$ rates of 95.84% and 95.14% respectively.

### Performance analysis by varying classifiers with t-Test

**Fig. 8:**
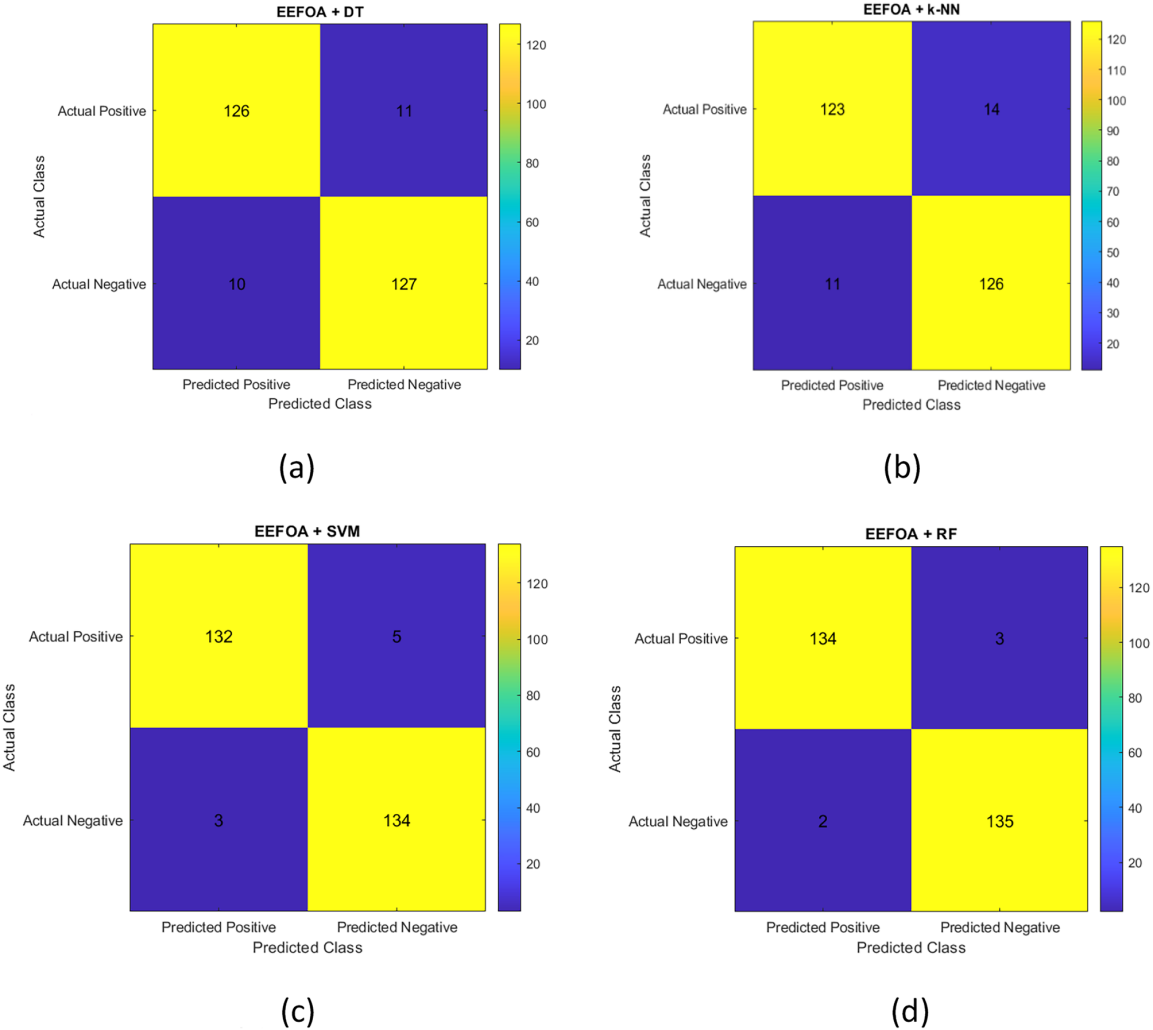
Confusion matrices of EEFOA in combination with different classifiers (**a**) DT (**b**) k-NN (**c**) SVM (**d**) RF.

**Fig. 9:**
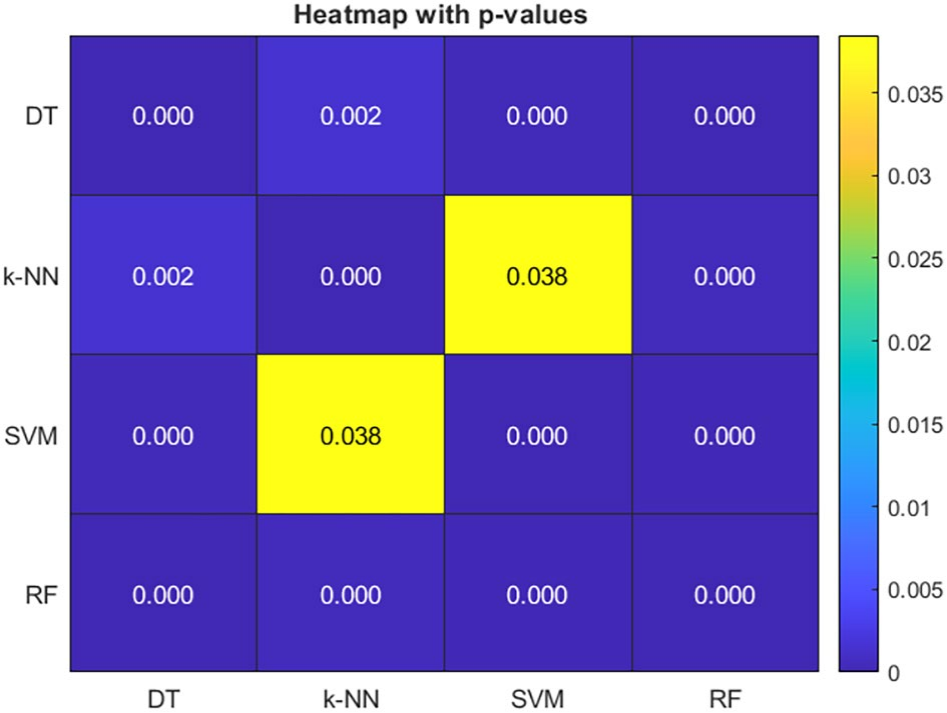
Heatmap visualization.

As stated earlier, different classifiers such as DT, k-NN, SVM and RF are employed upon different features and the performances are shown in Fig. 2. Based on the results, it is evident that RF outperforms other classifiers. To be even more specific, RF performs well with EEFOA and is followed by the combination of chi-square and ANOVA. SVM is the second best performer, when combined with EEFOA and XGBoost performs well with SVM. Hence, RF classifier with EEFOA feature selection technique performs well for the dataset. The confusion matrices of all the classifiers are shown in Fig. 8.

t-test is also carried out for the accuracy rates attained by four different classifiers such as Decision Tree (DT), k-Nearest Neighbour (k-NN), Support Vector Machine (SVM) and Random Forest (RF) with EEFOA based feature selection in ten folds. Figure 9 shows the heatmap of p-values obtained in pair-wise t-test and Table 3 shows the p-values.

**Table 3:** p-values of pairwise t-test.

Classifiers	DT	k-NN	SVM	RF
DT	0	0.0016125	0.00044551	0.0001213
k-NN	0.0016125	0	0.038475	0.00046743
SVM	0.00044551	0.038475	0	0.00025885
RF	0.0001213	0.00046743	0.00025885	0

From the results, it is evident that all classifiers show significant difference, except k-NN-SVM. However, DT, SVM and RF are significantly different. The standard deviations over 10 runs of all the classifiers are presented in Table 4 concerning accuracy and F1-score.

**Table 4:** SD in accuracy and F-measure.

Classifier	Mean (Accuracy) Rate	Standard Deviation (Accuracy)	Mean F1-score	Standard Deviation (F1-score)
DT	0.9452	0.019303	0.921	0.013703
k-NN	0.9682	0.01039	0.907	0.0082327
SVM	0.9783	0.0078888	0.9718	0.0090652
RF	**0.9879**	**0.0041753**	**0.9815**	**0.0062405**

**Fig. 10:**
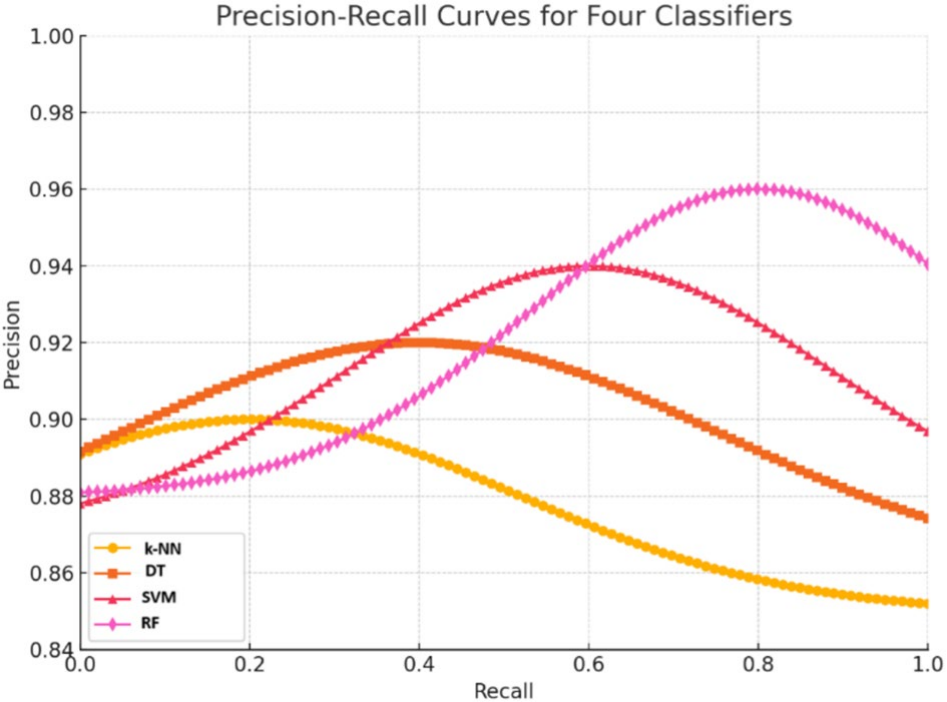
PR curve of different classifiers.

From the experimental results, the RF classifier performs better with greater mean accuracy and F1-score, while showing least SD that shows the consistency in performance. SVM is slightly variable, when compared to RF. Hence, RF is stable with better accuracy and F1-score rates. The PR curve of the classifiers is shown in Fig. 10.

Hence, the overall performance of the classification model is visualized and the false classification rates are easily figured out. The following section discusses the MCC values of the proposed work.

### MCC analysis

**Fig. 11:**
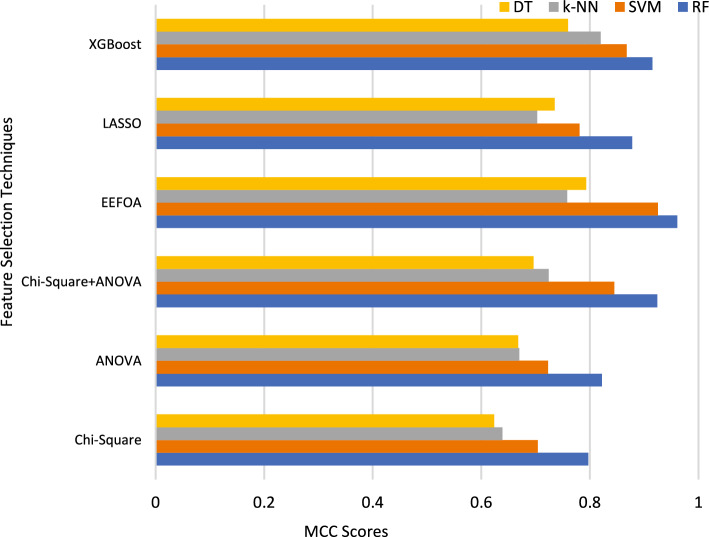
MCC analysis.

The quality of the binary classification model is checked by the MCC, as it considers all TP, TN, FP and FN values for analysis. The value of MCC ranges between -1 and 1, where the value ‘1’ indicates better classification and vice-versa. The MCC values of the proposed work is depicted in Fig. 11.

From Fig. 11, it is evident that the MCC score of nine techniques cross 0.8 and all the techniques fall within 0.8. XGBoost with k-NN, SVM and RF shows MCC rates of 0.82, 0.86 and 0.91 respectively. As far as LASSO is concerned, it performs well with RF classifier by showing an MCC of 0.878. EEFOA performs well wiyj SVM and RF by proving MCC scores of 0.925 and 0.961. The MCC score of ANOVA in combination with RF is 0.82.

### Computational complexity analysis of feature selection techniques

The computational complexities of different feature selection techniques are tabulated in Table 5.

**Table 5:** Computational complexity analysis of different feature selection techniques.

Technique	Type	Computational Complexity	Remarks
Chi-Square	Filter	$O(n\times m)$	Fast for continuous features
ANOVA	Filter	$O(n\times m)$	Fast for continuous features
LASSO	Embedded	$O(k\times n\times m)$/iteration	Requires iterative convergence
XGBoost	Embedded	$O(k1\times m\times \text{log}(n))$ per tree	Good accuracy rates
EEFOA	Wrapper	$O(p\times k\times c)$	Slow in speed, but most effective

Here, $n, m, k, k1, p and c$ represent samples, features, iterations, boosting round, population and classifier training cost respectively. Though EEFOA is the slowest of all, it proves the greatest accuracy rates.

### Performance comparison with existing works

The performance of the proposed work is compared with the existing works in terms of the above stated performance metrics and the results are tabulated in Table 6.

**Table 6:** Performance comparison with existing works.

Techniques	$\partial$(%)	$\wp$(%)	$\mathfrak{R}$(%)	$\mathcal{F}$(%)
ML framework ^25^	98	97	98	98
Prediction model ^26^	86.3	82.4	80.9	81.6
ML algorithms ^27^	96	96	95	95.49
Feature selection + ML ^28^	**98.8**	96.72	97.3	97
**EEFOA + RF**	**98.8**	**98.3**	**98.1**	**98.19**

The performance of the proposed work is better than the exsiting studies and it is notable that the accuracy rates of the proposed EEFOA + RF is equivalent to the work presented in^28^. However, all the comparative works consider open dataset from Kaggle with 541 records, whereas the proposed work considers 1368 records with better performance. Although the existing work in^28^ shows the same accuracy rate of 98.8%, the precision and recall rates are 96.7% and 97.3%, which means FP and FN rates are greater and so are the false alarms. The F-measure rate of^28^ is 97%, while the proposed work is 98.19%. The main reason for the better performance of the proposed model is the incorporation of effective pre-processing techniques along with the exploration of feature selection techniques. The ML model is trained by the most significant, which made it possible to classify between the classes.

### Discussion

All the comparative works attempted to classify between PCOS and Non-PCOS patients, which is carried out on a open dataset with 541 samples. This work stands out from the rest of works by including certain interesting features such as HOMA-IR, Sebarrhoea, LDL cholestrol level and Acne. The impact of a specific feature in determining PCOS/Non-PCOS is not analysed in any of the comparative works. Besides, the proposed work explores different feature selection approaches to arrive at an effective feature set, which makes the ML model efficient.

These features are significant in classifying between the PCOS and non-PCOS entities. The importance of vitamin D3 is studied and its impact is clearly defined in PCOS patients. The experimental analysis highlighted that the features Vitamin D3 and Homa-IR are associated, where Homa-IR is closely associated to PCOS. Our analysis show that the vitamin D3 deficiency/insufficiency is shown by 46% of PCOS patients. As a conclusion, Vitamin D3 supplementation could improve the symptoms confronted by the PCOS patients.

## Conclusions

This work presents a model that studies the impact of vitamin D3 over PCOS patients, while classifying the PCOS/Non-PCOS entities. A tailored dataset with 1041 records is built with 43 attributes, which is acquired from different websites and the final dataset is built by the advice obtained from physicians and Lab technicians. All the records are pre-processed to deal with missed values, outlier detection and data balancing by employing appropriate techniques. The feature selection process relies on different approaches such as filter based (chi-square,ANOVA), wrapper based (EEFOA) and embedded methods (LASSO,XGBoost). The so selected features is applied over ML models such as DT, k-NN, SVM and RF. A rigorous experimentation procedure is carried out and upon analysis, it is found that the performance of EEFOA with RF outperforms all the other combinations with the $\partial$ rates of 98.8%, the $\wp ,\mathfrak{R}$ and $\mathcal{F}$ rates of 98.3%, 98.1% and 98.19% respectively. In order to enhance interpretability, this work incorporates SHAP and LIME to emphasize the importance of features in PCOS classification. The paper is concluded with the remark that Vitamin D3 deficiency/insufficiency is strongly associated with the PCOS affected patients, while the impact is minimal in PCOS unaffected individuals. Hence, vitamin D3 supplementation could help in improving the ill-effects of PCOS. In future, this work is planned to be extended by considering other important feature, while PCOS classification is executed. External validations of this work can be carried out for analysing the generalization and clinical relevance.

## Data Availability

The data that support the findings of this study are available from the corresponding author, upon reasonable request.
